# Relationship between increased maternal serum free human chorionic gonadotropin levels in the second trimester and adverse pregnancy outcomes: a retrospective cohort study

**DOI:** 10.1186/s12905-024-03105-z

**Published:** 2024-06-04

**Authors:** Yiming Chen, Xiaoqing Dai, Bin Wu, Chen Jiang, Yixuan Yin

**Affiliations:** 1 Department of Prenatal Diagnosis and Screening Center, Hangzhou Women’s Hospital (Hangzhou Maternity and Child Health Care Hospital), Shangcheng District Hangzhou, No. 369, Kunpeng Road, Zhejiang, 310008 China; 2 School of Medical Technology and Information Engineering, Zhejiang Chinese Medical University, Hangzhou, 310053 China

**Keywords:** Maternal serum, Free beta-subunit human chorionic gonadotropin, Adverse pregnancy outcomes, Risk ratios, Retrospective cohort study

## Abstract

**Background:**

A retrospective cohort study was conducted to collect the data of pregnant women who received hospital delivery in Hangzhou Women's Hospital from January 2018 to December 2020, and who participated in the second trimester (15–20^+6^ weeks) of free beta human chorionic gonadotropin (free β-hCG). And the study was conducted to explore the relationship between maternal serum free β-hCG and adverse pregnancy outcomes (APO).

**Methods:**

We retrospectively analyzed the clinical data of 1,978 women in the elevated maternal serum free β-hCG group (free β-hCG ≥ 2.50 multiples of the median, MoM) and 20,767 women in the normal group (0.25 MoM ≤ free β-hCG < 2.50 MoM) from a total of 22,745 singleton pregnancies, and modified Poisson regression analysis was used to calculate risk ratios (RRs) and 95% confidence intervals (CI) of the two groups.

**Results:**

The gravidity and parity in the elevated free β-hCG group were lower, and the differences between the groups were statistically significant (all, *P* < 0.05). The risks of polyhydramnios, preeclampsia, and hyperlipidemia, were increased in women with elevated free β-hCG levels (RRs: 1.996, 95% CI: 1.322–3.014; 1.469, 95% CI: 1.130–1.911 and 1.257, 95% CI: 1.029–1.535, respectively, all *P* < 0.05), intrauterine growth restriction (IUGR) and female infants were also likely to happen (RRs = 1.641, 95% CI: 1.103–2.443 and 1.101, 95% CI: 1.011–1.198, both *P* < 0.05). Additionally, there was an association between elevated AFP and free β-hCG levels in second-trimester (RR = 1.211, 95% CI: 1.121–1.307, *P* < 0.001).

**Conclusions:**

APOs, such as polyhydramnios, preeclampsia, and hyperlipidemia, were increased risks of elevated free β-hCG levels, IUGR and female infants were also likely to happen. Furthermore, there was an association between elevated AFP levels and elevated free β-hCG levels in second-trimester. We recommend prenatal monitoring according to the elevated maternal serum free β-hCG level and the occurrence of APO.

**Supplementary Information:**

The online version contains supplementary material available at 10.1186/s12905-024-03105-z.

## Background

Human chorionic gonadotropin (hCG), a glycoprotein ranging in molecular weight from 36 to 41 kDa of poorly and highly glycosylated forms, is comprised of two non-covalently bound subunits. It has four major isoforms, namely, classical hCG, hyperglycosylated hCG, free β-subunit hCG, and sulfated hCG. This hormone is initially secreted by trophoblasts to promote the secretion of progesterone by the corpus luteum, support the coordinated growth and expansion of the embryo and uterus, mediate signaling in the endometrium, and support the growth and development of the umbilical cord as well as fetal organs [1, 2]. The hCG or free beta-subunit human chorionic gonadotropin (free β-hCG) is synthesized from early pregnancy until 15–20 weeks of pregnancy, after which its synthesis decreases until birth [3, 4].

Maternal serum free β-hCG is a biochemical indicator of aneuploidy, such as trisomy 21, 18 and 13, during second-trimester prenatal screening [5, 6]. There was no agreement on the relationship between serum free β-hCG levels and adverse pregnancy outcomes (APO). Ozdemir et al. [7] reported correlations of the levels of the first- and second-trimester serum markers plasma protein-A (PAPP-A) and alpha-fetoprotein (AFP) with intrauterine growth restriction (IUGR) and macrosomia. In the absence of medically-indicated risk factors for premature delivery, the elevated serum free β-hCG levels of first- and second-trimester pregnant women are associated with a reduced likelihood of spontaneous preterm birth [8]. Likewise, Parry et al. [9] suggested that serum levels, including free β-hCG, in early pregnancy were significantly associated with pregnancy outcomes; however, the assays used to test the analytes did not support their use as clinical biomarkers to predict APO, either alone or in combination with maternal characteristics. Furthermore, Tahe et al. [10] reported that there was a significant difference in β-hCG concentrations between women with preeclampsia and normotensive women, and there was no significant difference in perinatal or maternal outcomes between groups, except in women with β- hCG ≥ 40,000 mIU/mL.

There is an association between abnormal biochemical indicators detected during aneuploidy screening and adverse pregnancy outcomes such as preeclampsia and IUGR. In 2008, the Canadian Association of Obstetricians and Gynecologists made the following recommendations for screening in the second trimester of pregnancy: (i) unexplained maternal serum AFP (> 2.50 MoM), hCG (> 3.00 MoM) and/or inhibin A (≥ 2.00 MoM) levels; and (ii) increased or decreased maternal serum AFP (< 0.25 MoM) and/or unconjugated estriol (< 0.50 MoM) levels, all of which increase the risk of adverse pregnancy outcomes and no specific treatment plan at present [11], long obstetric follow-up is often required.

This retrospective cohort study, which based on the clinical data of 22,745 women in the second trimester of pregnancy (15–20^+6^ weeks), was conducted to determine the relationship between maternal serum free β-hCG levels and APO.

## Materials and methods

### Participants

**Fig. 1:**
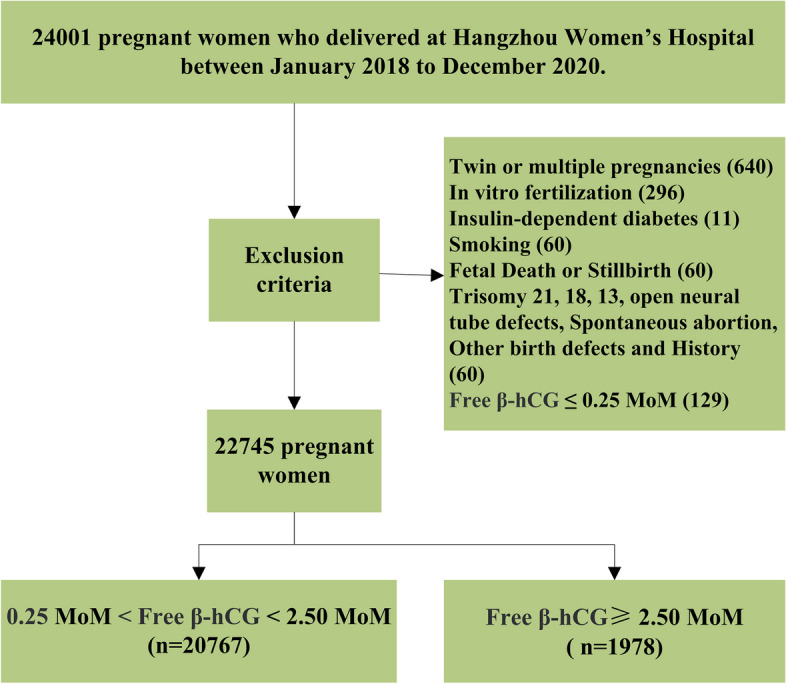
Flow chart of patient selection [12]

Using a retrospective cohort design, we collected data from 24,001 pregnant women who delivered at the Department of Obstetrics, Hangzhou Women’s Hospital, from January 2018 to December 2020, and participated in maternal serum AFP and free β-hCG screening during the second trimester (15–20^+6^ weeks). After excluding cases that failed to satisfy the inclusion criteria, a total of 22,608 cases were included, as shown in Fig. 1. Of these, 1,978 cases had elevated maternal serum free β-hCG levels [free β-hCG ≥ 2.50 multiples of the median (MoM)] and 20,767 cases had normal maternal serum free β-hCG levels (0.25 MoM < free β-hCG < 2.50 MoM). This study was approved by the Medical Ethics Committee of Hangzhou Women’s Hospital (2023–002). This research has obtained informed consent from the patients.

### Diagnostic and exclusion criteria

APO of complications: gestational hypertension, preeclampsia, gestational diabetes mellitus, intrahepatic cholestasis, thrombocytopenia, hyperlipidemia, arrhythmia, hypothyroidism/hyperthyroidism, premature rupture of membranes, fetal distress, IUGR, premature loss of placenta, oligohydramnios/polyhydramnios, umbilical cord around neck, premature delivery, low birth weight, and macrosomia. All pregnancy complications and pregnancy outcomes were obtained from clinical records diagnosed by obstetricians in hospitals according to the relevant Chinese guidelines [13–16]. Low birth weight referred to a neonatal weight < 2,500 g, whereas macrosomia referred to a newborn weighing > 4,000 g. Premature birth referred to birth days less than 238 days. Oligohydramnios was an amniotic fluid index (AFI) < 5 cm, while the AFI of polyhydramnios was more than 25 cm or a vertical pocket of at least 8 cm. IUGR referred to an estimated weight on ultrasonographic examination below the 10 th percentile adjusted to gestational age [17].

The exclusion criteria were as follows: twin or multiple gestations, in vitro fertilization pregnancies, a history of insulin-dependent diabetes, a history of maternal smoking, fetal death or stillbirth, chromosome abnormalities such as trisomy 18 or 21, fetal open neural tube defects and/or other congenital defects, and free β-hCG ≤ 0.25 MoM in pregnant women with incomplete data [12] (Fig. 1).

### Statistical analysis

Statistical analysis was performed with SPSS 21.0 software (IBM, Armonk, NY, USA). The Kolmogorov–Smirnov test was used to determine data normality, and skewed distribution was expressed as median and percentile [M (P_2.5_—P_97.5_)]. The Mann–Whitney U test or Chi-Squared test was used for univariate analysis of continuous or categorical data. *P* < 0.10 was selected as the screening criterion for modified Poisson regression analysis to calculate the risk ratios (RRs) and 95% confidence intervals (CIs) of the influencing factors. The variables were maternal weight, gestational day, AFP MoM, gravidity, parity, hypertension, hyperlipidemia, fetal distress, anemia, postpartum hemorrhage, abnormal amniotic fluid volume, IUGR, and low birth weight and macrosomia, and the main effect method was used to establish the statistical model. APO in pregnant women with elevated free β-hCG levels were assessed by RR. *P* < 0.05 was considered statistically significant.

### Patient and public involvement

Patients and/or the public were not involved in the design, or conduct, or reporting, or dissemination plans of this research.

## Results

### Basic demographic data

The levels of AFP MoM and free β-hCG MoM in women of the elevated serum free β-hCG group were higher than those in women of the normal serum free β-hCG group (1.13 vs. 1.03, 3.69 vs. 1.08, respectively), whereas gravidity and parity in the elevated serum free β-hCG group were lower than those in the normal serum free β-hCG group (46.30% vs. 49.10%, 27.50% vs. 32.40%, respectively), and the differences were statistically significant (all *P* < 0.05). No significant difference in maternal age, maternal weight, height, body mass index, gestational age, systolic blood pressure, diastolic blood pressure, mean arterial pressure, mode of delivery, infant weight, infant length, fetal score after birth, and fetal gender was detected between the elevated serum free β-hCG elevated group and the normal serum free β-hCG group (all *P* > 0.05), as shown in Tables 1 and 2.

**Table 1:** Univariate demographic analysis of pregnant in the elevated maternal serum free β-hCG group and normal group n (%)

Indicators	Groups		*Z/x*^*2*^	*P*-values
	free β-hCG normal group (*n* = 20,767)	elevated free β-hCG group (*n* = 1978)		
AFP MoM	1.03 (0.55–1.81)	1.13 (0.58–2.16)	10.528	< 0.001^*^
free β-hCG MoM	1.08 (0.36–2.28)	3.69 (2.52–7.98)	73.606	< 0.001^*^
Maternal age (years old)	29.00 (23.00–37.00)	29.00 (23.00–37.00)	0.235	0.814
Categories (Maternal age) (years old)			0.163	0.997
20–24.9	10,312 (49.66)	991 (50.10)		
25–29.9	1460 (7.03)	138 (6.98)		
30–34.9	7500 (36.11)	709 (35.84)		
35–39.9	1363 (6.56)	128 (6.47)		
≥ 40	132 (0.64)	12 (0.61)		
Maternal weight (kg)	67.00 (53.00–86.85)	67.00 (53.00–87.00)	1.035	0.300
Maternal height (cm)	160.00 (151.00–170.00)	160.00 (151.00–171.00)	0.153	0.878
BMI (kg/m^2^)	25.89 (21.09–32.81)	25.85 (20.96–33.04)	1.052	0.293
Categories (BMI)			1.661	0.646
Thin (< 18.5 kg/m^2^)	27 (0.14)	4 (0.20)		
Obese (25–29.9 kg/m^2^)	10,927 (52.62)	1035 (52.33)		
Obesity (≥ 30 kg/m^2^)	2154 (10.37)	193 (9.76)		
Normal (18.5 kg/m^2^-25 kg/m^2^)	7656 (36.87)	746 (37.71)		
Gestational age (days)	273.00 (252.00–287.00)	273.00 (249.45–287.00)	1.074	0.283
Categories (Gestational days)			5.027	0.081
< 259 days	900 (4.33)	106 (5.36)		
> 287 days	240 (1.16)	19 (0.96)		
Normal (259 days-287 days)	19,627 (94.51)	1853 (93.68)		
Systolic blood pressure (SBP) (mmHg)	118.00 (98.00–138.00)	118.00 (98.00–138.43)	0.548	0.584
Diastolic blood pressure (mmHg)	73.00 (60.00–91.00)	73.00 (60.00–95.00)	0.964	0.335
Arterial pressure difference (MAP) (mmHg)	87.33 (74.00–105.67)	87.67 (74.67–108.33)	0.779	0.436
Gravidity			5.783	0.016^**^
No(0)	10,573 (50.90)	1063 (53.70)		
Yes (≥ 1)	10,194 (49.10)	915 (46.30)		
Parity			20.501	< 0.001^*^
No(0)	14,034 (67.60)	1435 (72.50)		
Yes (≥ 1)	6733 (32.40)	543 (27.50)		
Mode of delivery			2.170	0.141
Vaginal delivery	14,432 (69.50)	1343 (67.90)		
Cesarean Section	6335 (30.50)	635 (32.10)		

Elevated free β-hCG group, 0.25 < free β-hCG MoM < 2.50; free β-hCG normal group, free β-hCG MoM ≥ 2.50

*AFP* alpha-fetoproteins,* free β-hCG* free beta-subunit human chorionic gonadotropin, *MoM* multiples of the median, *BMI* Body Mass Index, *SBP* systolic blood pressure, *BP* Blood Pressure, *MAP* maternal mean arterial pressure

^*^P < 0.001

^**^P < 0.05

**Table 2:** Univariate demographic analysis of newborn in the elevated maternal serum free β-hCG and normal groups n (%)

Indicators	Groups		*Z/x*^*2*^	*P*-values
	Free β-hCG normal group (*n* = 20,767)	Elevated free β-hCG group (*n* = 1978)		
Infant weight	3300.00 (2450.00–4108.25)	3300.00 (2194.75–4140.00)	0.654	0.513
Categories (Infant weight)			18.264	< 0.001^*^
Low birth weight infants (< 2500 g)	594 (2.86)	90 (4.55)		
Fetal macrosomia (≥ 4000 g)	813 (3.91)	83 (4.20)		
Normal (2500 g-4000 g)	19,360 (93.22)	1805 (91.25)		
Infant length	50.00 (48.00–51.00)	50.00 (47.00–51.00)	1.336	0.182
Apgar scores	10.00 (9.00–10.00)	10.00 (9.00–10.00)	1.360	0.174
Infant gender			3.803	0.149
Female	10,874 (52.36)	991 (50.10)		
Male	9893 (47.64)	987 (49.90)		

Elevated free β-hCG group, 0.25 < free β-hCG MoM < 2.50; free β-hCG normal group, free β-hCG MoM ≥ 2.05

*Free β-hCG* free beta-subunit human chorionic gonadotropin, *MoM* multiples of the median

^*^P < 0.001

^**^P < 0.05

### Univariate analysis of related pregnancy complications

Univariate analysis showed that hypertension, hyperlipidemia, anemia, abnormal amniotic fluid volume, fetal distress, postpartum hemorrhage, and IUGR and related categories (infant weight) were correlated with elevated serum free β-hCG levels (all *P* < 0.10). No significant difference was observed in the risk of influencing factors between the elevated serum free β-hCG MoM group and the normal serum free β-hCG group (all *P* > 0.10, Tables 3 and 4).

**Table 3:** Pregnancy complications of mothers in the elevated maternal serum free β-hCG group and normal group n (%)

Indicators	Groups		*Z/x*^*2*^	*P*-values
	Free β-hCG normal group	Elevated free β-hCG group		
	(*n* = 20,767)	(*n* = 1978)		
Hypertensive disorders of pregnancy (HDP)			14.316	< 0.001^*^
Preeclampsia	299 (1.44)	50 (2.53)		
Gestational hypertension	717 (3.45)	71 (3.69)		
Normal blood pressure	19,751 (95.11)	1857 (93.88)		
Intrahepatic cholestasis of pregnancy			0.035	0.853
No	20,401 (98.24)	1942 (98.18)		
Yes	366 (1.76)	36 (1.82)		
Hyperlipidaemia			5.181	0.023^**^
No	20,021 (96.41)	1887 (95.40)		
Yes	746 (3.59)	91 (4.60)		
Cesarean scar			0.508	0.476
No	18,260 (87.93)	1750 (88.47)		
Yes	2507 (12.07)	228 (11.53)		
Arrhythmias			1.217	0.270
No	20,703 (99.69)	1969 (99.54)		
Yes	64 (0.31)	9 (0.46)		
Gestational diabetes mellitus			0.232	0.630
No	17,981 (86.58)	1705 (86.20)		
Yes	2786 (13.42)	273 (13.80)		
Thyroid function			0.063	0.969
Hyperthyroidism	45 (0.22)	4 (0.20)		
Hypothyroidism	2511 (12.09)	236 (11.93)		
Normal thyroid function	18,211 (87.69)	1738 (87.87)		
Pregnancy with anemia			3.886	0.049^**^
No	15,834 (76.25)	1469 (74.27)		
Yes	4933 (23.75)	509 (25.73)		
Thrombocytopenia			0.725	0.395
No	20,500 (98.71)	1957 (98.94)		
Yes	267 (1.29)	21 (1.06)		
Uterine Inertia			1.963	0.161
No	20,014 (96.37)	1894 (95.75)		
Yes	753 (3.63)	84 (4.25)		
Amniotic fluid volume			9.981	0.007
Polyhydramnios	92 (0.44)	19 (0.96)		
Oligohydramnios	1113 (5.36)	104 (5.26)		
Normal amniotic fluid volume	19,562 (94.20)	1855 (93.78)		
Placenta previa			0.738	0.390
No	20,633 (99.35)	1962 (99.19)		
Yes	134 (0.65)	16 (0.81)		
Placental abruption			0.154	0.695
No	20,665 (99.51)	1967 (99.44)		
Yes	102 (0.49)	11 (0.56)		

Elevated free β-hCG group, 0.25 < free β-hCG MoM < 2.50; free β-hCG normal group, free β-hCG MoM ≥ 2.50

*Free β-hCG* free beta-subunit human chorionic gonadotropin, *MoM* multiples of the median

^*^P < 0.001

^**^P < 0.05

**Table 4:** Pregnancy outcomes of mothers in the elevated maternal serum AFP group and normal group n (%)

Indicators	Groups		*Z/x*^*2*^	*P*-values
	Free β-hCG normal group (*n* = 20,767)	Elevated free β-hCG group (*n* = 1978)		
Fetal distress			2.986	0.084
No	18,830 (90.67)	1770 (89.48)		
Yes	1937 (9.33)	208 (10.52)		
Postpartum hemorrhage			3.836	0.050
No	20,707 (99.71)	1977 (99.95)		
Yes	60 (0.29)	1 (0.05)		
Premature rupture of menbranes			0.949	0.330
No	15,960 (76.85)	1501 (75.88)		
Yes	4807 (23.15)	477 (24.12)		
Cord entanglement			1.844	0.174
No	14,329 (69.00)	1394 (70.48)		
Yes	6438 (31.00)	584 (29.52)		
Cesarean delivery			1.979	0.159
No	14,438 (69.52)	1345 (68.00)		
Yes	6329 (30.48)	633 (32.00)		
IUGR			20.763	< 0.001^*^
No	20,680 (99.58)	1955 (98.84)		
Yes	87 (0.42)	23 (1.16)		

Elevated free β-hCG group, 0.25 < free β-hCG MoM < 2.50; free β-hCG normal group, free β-hCG MoM ≥ 2.50

*Free β-hCG* free beta-subunit human chorionic gonadotropin, *MoM* multiples of the median, *IUGR* intrauterine growth retardation

^*^P < 0.001

^**^P < 0.05

### Modified Poisson regression analysis

As shown in Table 5, the risk of pregnancy complications, such as polyhydramnios, preeclampsia, and hyperlipidemia, was increased in women with elevated serum free β-hCG levels, with RRs of 1.996, 95% CI: 1.322–3.014; 1.469, 95% CI: 1.130–1.911 and 1.257, 95% CI: 1.029–1.535, respectively (all *P* < 0.05), and women with elevated serum free β-hCG levels were more likely to have IUGR and female infants, with RRs of 1.641, 95% CI: 1.103–2.443) and 1.101, 95% CI: 1.011–1.198) (both *P* < 0.05). Additionally, maternal characteristics, such as elevated AFP levels, were correlated with elevated β-hCG levels, with an RR of 1.211, 95% CI: 1.121–1.307, *P* < 0.001.

**Table 5:** Modified Poisson regression analysis of maternal characteristics and pregnancy outcomes

Indicators	β	Standard error	Wald	df	*P*-values	RR	95% CI for RR
(intercept)	-1.249	0.5161	5.858	1	0.016^**^	0.287	0.104–0.789
Gravidity							
≥ 1	0.048	0.0572	0.690	1	0.406	1.049	0.937–1.173
= 0^a^						1	
Parity							
≥ 1	-0.243	0.0646	14.189	1	< 0.001^*^	0.784	0.691–0.890
= 0^a^						1	
HDP							
Preeclampsia	0.385	0.1341	8.226	1	0.004^**^	1.469	1.130–1.911
Gestational hypertension	0.048	0.1159	0.170	1	0.680	1.049	0.836–1.316
Normal blood pressure^a^						1	
Hyperlipidaemia							
Yes	0.229	0.102	5.019	1	0.025^**^	1.257	1.029–1.535
No^a^						1	
Fetal distress							
Yes	0.041	0.0721	0.323	1	0.570	1.042	0.905–1.20
No^a^						1	
Pregnancy with anemia							
Yes	0.087	0.0491	3.117	1	0.077	1.091	0.991–1.201
No^a^						1	
Amniotic fluid volume							
Polyhydramnios	0.691	0.2103	10.800	1	0.001^**^	1.996	1.322–3.014
Oligohydramnios	-0.068	0.0972	0.486	1	0.486	0.934	0.772–1.131
Normal amniotic fluid volume^a^						1	
IUGR							
Yes	0.496	0.2028	5.971	1	0.015^**^	1.641	1.103–2.443
No^a^						1	
Infant weight (g)							
≥ 4000 g	0.113	0.1098	1.056	1	0.304	1.119	0.903–1.388
< 2500 g	0.224	0.1325	2.872	1	0.090	1.252	0.965–1.623
2500 g-4000 g^a^						1	
Gestational days							
> 287 days	-0.224	0.2229	1.009	1	0.315	0.799	0.516–1.237
< 259 days	0.041	0.1137	0.127	1	0.721	1.041	0.833–1.301
287—259 days^a^						1	
Infant gender							
Female	0.096	0.0432	4.948	1	0.026^**^	1.101	1.011–1.198
Male^a^						1	
Maternal weight	-0.005	0.0029	2.643	1	0.104	0.995	0.990–1.001
Gestational days	-0.01	0.0041	6.015	1	0.014^**^	0.990	0.982–0.998
AFP MoM	0.191	0.0392	23.798	1	< 0.001^*^	1.211	1.121–1.307

Model: (intercept), gravidity, parity, HDP (gestational hypertension, preeclampsia), Hyperlipidaemia, Fetal distress, Pregnancy with anemia, Amniotic fluid volume, IUGR, Infant gender, Maternal weight, Gestational days, AFP MOM

*HDP* hypertensive disorders of pregnancy, *IUGR* intrauterine growth retardation, *AFP* alpha-fetoproteins, *MoM* multiples of the median, *RR* relative risk, *CI* confidence intervals

^*^P < 0.001

^**^P < 0.05

^a^Reference

## Discussion

In this study, the pregnancy outcomes of 1978 s-trimester pregnant women with serum free β-hCG ≥ 2.50 MoM and 20,767 s-trimester pregnant women with serum free β-hCG 0.25 MoM < free β-hCG < 2.50 MoM in Hangzhou, China, were analyzed. The results indicated that the risk of pregnancy complications, such as polyhydramnios, preeclampsia, and hyperlipidemia, was increased in women with elevated serum free β-hCG levels, and elevated serum free β-hCG levels were related to APO such as IUGR. Additionally, there was an association between elevated AFP levels and elevated β-hCG levels in the sera of pregnant women.

The results of this study showed that polyhydramnios (RR: 1.996, 95% CI: 1.322–3.014) was higher in pregnant women with high serum free β-hCG levels than that in pregnant women with normal levels of the hormone, consistent with previous studies that reported a correlation of second-trimester non-immune hydrops (including hydramnios) with elevated maternal hCG levels [18] as well as a correlation of placental chorioangioma with early severe polyhydramnios and elevated maternal serum hCG levels [19]. In China, researchers have used proteomic techniques to identify many biomarkers for pregnancy-related pathological conditions, such as β-hCG for Down's syndrome and AFP for trisomy 13,18 syndrome, which were almost derived from or secreted by fetal, maternal circulation, and placental tissues [20]. The cohort study of Tashfeen et al*.* [21] showed that polyhydramnios could increase the risk of adverse perinatal outcomes, and polyhydramnios was positively correlated with maternal age, diabetes, fetal malformation and macrosomia. Pregnant women with high serum levels of free β-hCG were more likely to give birth to female infants (RR: 1.101, 95% CI: 1.011–1.198), which was similar to the results of a recent review report [22], which pointed out that pregnant women with female fetuses have higher serum β-hCG levels than those with male fetuses.

Free β-hCG is produced by the trophoblast layer outside the villi, and syncytiotrophoblast cells are the main placental source of free β-hCG. The main function of free β-hCG is to promote the synthesis of progesterone in the corpus luteum, maintain the resting state of the uterine muscle layer and pregnancy, until the placenta itself ensures the production of progesterone [23]. The exact time and form of free β-hCG secretion peak in early pregnancy are still not reasonably explained. Placental ischemic diseases such as preeclampsia or IUGR are characterized by abnormal invasion of trophoblasts, which can lead to reduced uterine placental blood flow (detected by Doppler ultrasound) and uterine placental ischemia, resulting in excessive or insufficient production of various pro angiogenic and antiangiogenic factors, which in turn affect the clearance rate or biological activity of free β-hCG, leading to high concentrations of free β-hCG [24, 25]. The results of our study demonstrated that preeclampsia was a risk for APO in pregnant women with elevated serum free β-hCG levels (RR: 1.469, 95% CI: 1.130–1.911), consistent with 2008 SOGC guidelines [11], which stated that hCG > 3.00 MoM was associated with an increased prevalence of APO. However, the guidelines failed to propose a relevant treatment plan. Tancrede et al. [26] confirmed that serum free β-hCG > 2.00 MoM was associated with a risk of delivery before term (RR: 4.60, 95% CI: 2.30–9.10], whereas serum free β- hCG > 2.00 MoM had no effects on some pregnancy outcomes (such as preeclampsia, IUGR, fetal death) after term (RR: 1.10, 95% CI: 0.70–1.70). These findings agree with those of another study in which no differences in pregnancy outcomes between women with serum free β-hCG > 2.00 MoM and those with normal levels of the hormone were reported, although the risks of preeclampsia and low birth weight were higher in the elevated group (cut-off level of incidence, 2.5) than in the control group [27]. This study also showed that IUGR is also a placenta-related factor (RR: 1.641, 95% CI: 1.103–2.443). This was similar to the research findings of Kiyokoba et al. [28], who also pointed out that IUGR and preeclampsia (PE) with IUGR (PE/IUGR) were high-risk perinatal diseases that might involve high levels of hCG and mitochondrial dysfunction, it could also elevated expression of β-hCG and growth differentiation factor mRNA and protein levels in the placenta with both diseases, which played an important role in the pathogenesis of IUGR and PE/IUGR.

The pregnant women were divided into two groups according to second-trimester biomarkers: the high-risk group [patients with AFP and/or hCG above the 75th percentile; odds ratio (OR) of pathological placentation: 2.27] and the low-risk group (patients with AFP and/or hCG below the 25th percentile; OR for pathological placentation: 0.38) [29]. High levels of AFP, free β-hCG, and inhibin A but a low level of unconjugated estriol significantly increased the risk of small for gestational age fetuses [30]. The incidence of APO, such as premature delivery, stillbirth, limb deformities, and chromosomal abnormalities in the maternal serum free β-hCG ≥ 2.00 MoM group, was significantly higher than that in the control group [31, 32], whereas the risk of gestational hypertension (OR: 1.72), premature delivery (OR: 2.50), and stillbirth (OR: 3.93) were increased in the elevated maternal serum β-hCG group [33].

The results of this study revealed an association between hyperlipidemia (RR: 1.257, 95% CI: 1.029–1.535) and elevated maternal serum free β-hCG levels, but few studies support this finding. Only in a previous report mentioned that the concentration of free β-hCG in postmenopausal women was much higher than that in premenopausal women (*P* < 0.001). Correspondingly, triglyceride (TG) was elevated (*P* < 0.05) [34]. Therefore, the relationship between hyperlipidemia and maternal serum free β-hCG requires further study.

The results of this study also suggested a correlation between elevated AFP levels (RR: 1.211, 95% CI: 1.121–1.307) and elevated β-hCG levels in second-trimester pregnant women. Chandra et al. [35] reported that, in addition to placental abruption, unexplained elevated maternal serum AFP or hCG levels were independently associated with pregnancy outcomes in both high risk and low risk women, and the RR of fetal death was high (in high risk and low risk women, the RR of AFP or hCG was 4.90, 95%CI: 2.70–8.70), suggesting that antenatal monitoring of patients with elevated AFP and/or hCG levels should be conducted regardless of prenatal risk status.

A previous study has reported that the association between placental-related histopathological changes and pregnancy complications was weak due to unexplained elevated maternal serum AFP and hCG levels [36]. Doppler ultrasound scans of the uterine artery and morphological examinations of the placenta revealed that most biochemical abnormalities in pregnant women resulted in premature delivery and/or perinatal death [37], which led us to recommend placental ultrasound for women with abnormal biochemical indicators when no fetal abnormality is found.A recent report showed that maternal serum free β-hCG concentration was associated with chromosomal structural abnormalities, When the maternal serum free β-hCG ≥ 5.00 MoM, the relative risk of trisomy 21 was higher. When free β-hCG < 0.20 MoM, the probability of trisomy 13, trisomy 18 and Turner syndrome also increased. Therefore, Doppler ultrasonography combined with the concentration of maternal serum free β-hCG can monitor fetal development to some extent [38].

In this study, we retrospectively analyzed APO, such as IUGR and preeclampsia, caused by elevated serum free β-hCG levels in the second trimester. However, there were several limitations. First, this study only included pregnancy outcomes of singleton-pregnant women conceived naturally in Hangzhou, China, from 2018 to 2020. Second, this study explored the relationship between pregnancy outcomes, such as IUGR and preeclampsia with elevated serum free β-hCG levels, and failed to investigate how the physiological mechanisms of IUGR and preeclampsia were affected, such as placental related disease mechanisms and abnormal umbilical artery diastolic blood flow. Lastly, additional studies of longer follow-up periods and larger sample sizes are needed.

## Conclusions

In conclusion, this study demonstrated that elevated maternal serum free β-hCG levels were associated with pregnancy complications such as polyhydramnios, preeclampsia, and hyperlipidemia. It is worth noting that the risk of APO could be monitored by the high level of maternal serum free β-hCG during the second trimester.

### Supplementary Information

**Supplementary Material 1.**

## Data Availability

All data generated or analyzed during this study are included in this published article and its supplementary information files.
